# Low Frequency of *ESRRA–C11orf20* Fusion Gene in Ovarian Carcinomas

**DOI:** 10.1371/journal.pbio.1001784

**Published:** 2014-02-04

**Authors:** Francesca Micci, Ioannis Panagopoulos, Jim Thorsen, Ben Davidson, Claes Gøran Tropé, Sverre Heim

**Affiliations:** 1 Section for Cancer Cytogenetics, Institute for Cancer Genetics and Informatics, The Norwegian Radium Hospital, Oslo University Hospital, Oslo, Norway; 2 Centre for Cancer Biomedicine, University of Oslo, Oslo, Norway; 3 Department of Pathology, The Norwegian Radium Hospital, Oslo University Hospital, Oslo, Norway; 4 Faculty of Medicine, University of Oslo, Oslo, Norway; 5 Department of Gynecology, The Norwegian Radium Hospital, Oslo University Hospital, Oslo, Norway; Medical Research Council, United Kingdom

## Abstract

The identification of recurrent gene fusions in common epithelial cancers—for example, *TMPRSS2/ERG* in prostate cancer and *EML4/ALK* in nonsmall cell lung carcinomas—has raised the question of whether fusion genes are pathogenetically important also in ovarian carcinomas. The first recurrent fusion transcript in serous ovarian carcinomas was reported by Salzman et al. in 2011, who used deep paired-end sequencing to detect the fusion gene *ESRRA–C11orf20* in 10 out of 67 (15%) serous ovarian carcinomas examined, a finding that holds great promise for our understanding of ovarian tumorigenesis as well as, potentially, for new treatment strategies. We wanted to test how frequent the *ESRRA/C11orf20* fusion is in ovarian carcinomas of all subtypes, and therefore examined a series of 230 ovarian carcinomas of which 197 were of the serous subtype and 163 of the 197 were of stages III and IV—that is, the very same carcinoma subset where the fusion transcript had been found. We performed PCR and high-throughput sequencing analyses in search of the fusion transcript. We used the same primers described previously for the detection of the fusion and the same primer combination, but found no *ESRRA/C11orf20* fusion in our series. A synthetic DNA plasmid containing the reported *ESRRA/C11orf20* fusion was included as a positive control for our PCR experiments. Data from high-throughput sequencing of 23 ovarian carcinomas were screened in search of alternative partner(s) for the *ESRRA* and/or *C11orf20* gene, but none was found. We conclude that the frequency of the *ESRRA/C11orf20* gene fusion in serous ovarian carcinomas of stages III and IV must be considerable less than that reported previously (0/163 in our experience compared with 10/67 in the previous study). At the very least, it seems clear that the said fusion cannot be a common pathogenetic event in this tumor type.

## Abstract 2

A study by Francesca Micci and colleagues shows that the ESRRA-C11orf20 fusion transcript is rare in ovarian carcinoma, contrary to a previously reported frequency of 15%.

## Author Summary

The identification of characteristic fusion genes in cancer helps us to understand how a particular cancer arises and also to improve classification and diagnosis, with a view to develop specific medical treatments that target exactly those aberrant molecules that trigger the disease. A fusion transcript presumed to arise from a chromosomal rearrangement involving the ESRRA and C11orf20 genes has previously been described to be present in 15% of serous ovarian carcinomas—the first fusion transcript to be associated with this common and often fatal cancer. We assessed 163 similar ovarian carcinomas for the presence of the ESRRA–C11orf20 fusion transcript, plus a further 67 ovarian carcinomas of different histologic subtypes/grades, to see if these tumors were characterized by the same fusion. Surprisingly, we found no ESRRA–C11orf20 transcripts in any of the 230 carcinomas. The question as to whether fusion genes contribute to the pathogenesis of ovarian carcinoma therefore remains open.

## Introduction

Cancer of the ovary makes up 30% of all malignant diseases of the female genital tract. Prognosis is poor, with a mean 5-year survival rate in Europe of 32%. This unfavorable outcome is largely attributable to a lack of early warning symptoms and signs and also a lack of diagnostic tests that allow early detection. As a result, approximately 70% of patients present with advanced stage, metastatic disease [1].

A number of specific genes have been identified as playing a role in ovarian carcinogenesis; the ones that have received the most attention are *BRCA1* and *BRCA2* followed by *TP53*. In addition, integrated genomic analysis of ovarian carcinomas has identified four ovarian cancer transcriptional subtypes, three microRNA subtypes, four promoter methylation subtypes, and a transcriptional signature associated with survival duration [2], attesting to the genetic complexity of these tumors.

The identification of recurrent gene fusions in common epithelial cancers—for example, *TMPRSS2/ERG* in prostate cancer [3] and *EML4/ALK* in nonsmall cell lung carcinomas [4],[5]—has raised the question of whether fusion genes are pathogenetically important also in ovarian carcinomas. Salzman et al. [6] reported the first recurrent fusion transcript in serous ovarian carcinomas. They used deep paired-end sequencing to detect the fusion gene *ESRRA–C11orf20* in 10 out of 67 (15%) serous ovarian carcinomas examined, a finding that holds great promise for our understanding of ovarian tumorigenesis as well as, potentially, for new treatment strategies. The fusion was brought about by rearrangements in the long arm of chromosome 11, in subband 11q13.1. The gene *ESRRA* (estrogen-related receptor alpha) encodes a nuclear receptor that is closely related to the estrogen receptor, whereas its partner is but an open reading frame sequence. Because *ESRRA* and *C11orf20* (also known as *TEX40*) normally lie only 11 kb apart, it is possible that the rearrangement leading to their fusion is an incidental consequence of another functionally important genetic event or that it is merely a “passenger” to other structural rearrangements.

To test how frequent *ESRRA/C11orf20* fusion is in ovarian carcinomas of all subtypes, we performed PCR analysis of 230 ovarian carcinomas, of which 197 were of the serous subtype and 163 of the 197 were of stages III and IV—that is, the very same carcinoma subset examined by Salzman et al. [6].

## Results and Discussion

**Figure 1:**
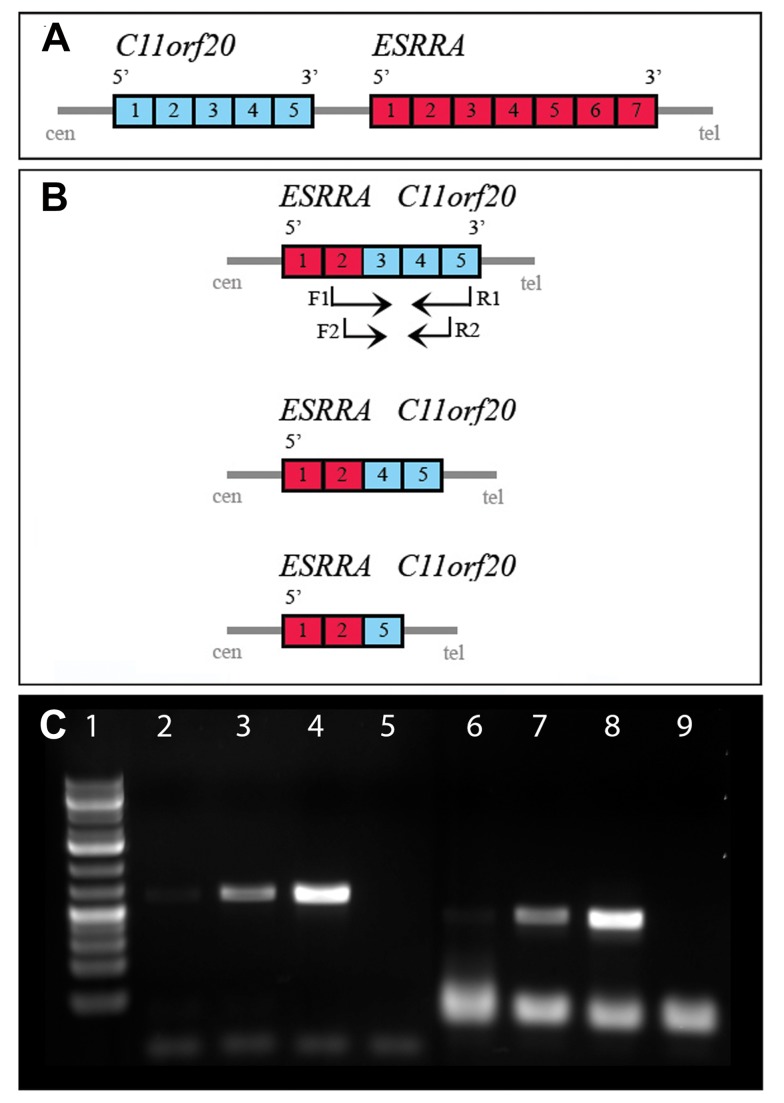
Putative *ESRRA-C11orf20* fusion transcript in ovarian carcinomas. (A) Schematic orientation of the *ESRRA* and *C11orf20* genes in the genome (the size of each exon is not in scale). (B) Representation of the three forms of the putative fusion with involvement of *ESRRA* exon 2 with *C11orf20* exon 3, 4, or 5. Arrows indicate position of the primers for the first PCR (F1 and R1) and the NESTED PCR (F2 and R2). Primer details are given in Materials and Methods. (C) Image of the gel electrophoresis for the synthetic transcript containing the fusion (internal positive control). A plasmid containing exon 2 of *ESRRA* and exon 5 of *C11orf20* was used as a positive control for our PCR experiments. Three different concentrations of the plasmid were tested using the *ESRRA* and *C11orf20* PCR primers. Lane 1, 1 kb DNA ladder; lanes 2 and 6, 2 fg plasmid (∼30 copies of the plasmid); lanes 3 and 7, 20 fg plasmid (∼300 copies of the plasmid); lane 4 and 8, 200 fg plasmid (∼3,000 copies of the plasmid). Lanes 2–5, primers G1P1-FWD (*ESRRA*) and REV_pair3 (*C11orf20*) used; lanes 6–9, inner (nested) primers G1P2-FWD (*ESRRA*) and F1-REV (*C11orf20*) used. Lanes 5 and 9 are negative controls with no plasmid template.

The PCR analysis of the 230 ovarian carcinomas showed no fusion transcript for the *ESRRA/C11orf20*. A synthetic DNA plasmid containing the reported *ESRRA/C11orf20* fusion was included as a positive control for our PCR experiments and was the only sample showing the transcript and demonstrating, at the same time, the validity of the experiments (Figure 1).

We also performed high-throughput sequencing of 23 ovarian carcinomas (already tested by PCR analysis), of which 10 were serous, five endometrioid, four clear cell, three mucinous, and one of a mixed endometrioid and undifferentiated subtype. Each sample was sequenced to yield about 60∼70 million reads using the Illumina HiSeq 2000 instrument. We extracted from the raw data all sequences containing the last 20 bp before the putative break of the *ESRRA* exon 2 gene sequence, getting 2,705 reads in total. We also found 58, 59, and 49 reads containing the first 20 bp of the *C11orf20* exon 3, exon 4, and exon 5 gene sequences, respectively (Table 1). From the extracted *ESRRA*- and *C11orf20*-specific sequences, none contained sequences of both *ESRRA* and *C11orf20*. The comparison was performed by investigating if the *ESRRA*-specific sequences contained *C11orf20* exon 3, 4, or 5 sequences and vice versa. It is possible to argue that the fusion gene, if present, should be driven by the *ESRRA* promoter, and therefore that the fusion gene read counts should be more similar to the high *ESRRA* ones than to the low *C11orf20* ones. As a result, assuming the presence of the fusion, the *C11orf20* reads should have been totally dominated by the fusion, something that was not seen (Table 1). Furthermore, all 2,705 sequences were used in a Blast search to verify their identity. The Blast search identified specific *ESRRA* and *C110rf20* sequences but revealed no sequences containing both *ESRRA* and *C11orf20* gene sequences. When searching in the same series of sequenced carcinomas (*n* = 23) for involvement of either the *ESRRA* or *C11orf20* in alternative fusions—that is, with other partner(s)—none was found.

**Table 1:** ESRRA and C11orf20 exon reads in high-throughput data files.

Gene	Exon	Number of Reads
ESRRA	Exon 2	2,705
C11orf20	Exon 3	58
C11orf20	Exon 4	59
C11orf20	Exon 5	49

We therefore conclude that the frequency of the *ESRRA/C11orf20* gene fusion in serous ovarian carcinomas of stages III and IV must be considerable less than that reported by Salzman et al. (0/163 in our experience, compared with 10/67 in their study) [6]. We have no explanation for the frequency differences observed. It is important to note that the difference in frequency calculated above is based only on adenocarcinomas of stages III and IV—that is, 163 tumors—as the remaining 67 tumors were of different histological subtypes or serous adenocarcinomas of grades I and II, which would not necessarily be expected to carry the same genetic fusion.

Looking into the possible mechanism—that is, chromosomal rearrangement(s)—by which the *ESRRA/C11orf20* fusion could have originated, it seems that a simple deletion or inversion could not alone have produced it. Both genes are located 5′ to 3′ from centromere to telomere on 11q, with *C11orf20* proximal to *ESRRA* (Figure 1); therefore, to get a fusion in which *ESRRA* is 5′ in a chimeric transcript would require a tandem duplication with a breakpoint in the central region. Regardless of how it may have been generated, however, it seems clear that the said fusion cannot be a common pathogenetic event in this tumor type.

## Materials and Methods

### Specimen Collection and RNA Extraction

The tumors were surgically removed at The Norwegian Radium Hospital from 1999 to 2010. The RNA was extracted using Trizol reagent according to the manufacturer's instructions (Invitrogen, Grand Island, NY), and its quality was checked by Experion Automated Electrophoresis System (Bio-Rad Laboratories, Hercules, CA). cDNA was synthesized using the Iscript advanced cDNA synthesis kit for RT-qPCR (Bio-Rad). Quality was checked using the TaqMan Gene Expression Assays for actin B (ACTB) and Glyceraldehyde 3-phosphate dehydrogenase (GAPDH). To measure the expression of ACTB and GAPDH, the assays Hs99999903_m1 and Hs99999905_m1, obtained from Applied Biosystems (Life Technologies, Carlsbad, CA), were used and run on a CFX96 Real-Time System (Bio-Rad).

### RT-PCR

For the first RT-PCR reaction we used the G1P1-FWD (5′-GGCATTGAGCCTCTCTACATCA-3′) mapping between 240 and 261 bp in the *ESRRA* gene (accession number NM_004451 version 4) and REV_pair3 (5′-GGGTCAGGCTTGGGTCTG -3′) located between 681 and 698 bp of the *C11orf20* (accession number NM_001039496 version 1) combination of primers—that is, the same primers as Salzman et al. [6]. The PCR cycles were as follows: initial denaturation at 94°C for 30 s, followed by 30 cycles, 15 s at 94°C, 30 s at 55°C; and 60 s at 70°C [6]. For the nested RT-PCR, the primers were G1P2-FWD (5′-AAAGGGTTCCTCGGAGACAGAGA-3′) located between 290 and 312 base pairs in the *ESRRA* gene (accession number NM_004451 version 4) and F1-REV (5′-TAATTCACGTACAGCCTCTTGCTCCG-3′) mapping between 597 and 622 bp of the *C11orf20* gene (accession number NM_001039496 version 1) [6]. The cycles were as follows: 15 s at 94°C, 30 s at 55°C, and 60 s at 72°C [6]. The nested PCR was run for 30 cycles. A synthetic DNA plasmid containing the reported *ESRRA/C11orf20* fusion was included as a positive control in our PCR experiments.

### RNA Sequencing

High-throughput sequencing was performed on 23 ovarian carcinomas, of which 10 were serous, five endometrioid, four clear cell, three mucinous, and one of a mixed endometrioid and undifferentiated subtype. A total of 3 µg of RNA was sent for high-throughput pair-end RNA-sequencing to the Norwegian Sequencing Centre at Ullevål Hospital (www.sequencing.uio.no). We used paired-end HTS with an average sequence read of 60–70 millions. We analyzed the sequences only with respect to the *ESRRA* and *C11orf20* genes. The last 20 bp of the *ESRRA* exon 2 gene sequence before the putative break and the first 20 bp of the *C11orf20* exon 3, exon 4, and exon 5 gene sequences have been extracted from the raw data (fastq-files) and further analyzed for putative gene fusions.

## Abbreviations

HTS: high-throughput sequencing
PCR: polymerase chain reaction
RT-PCR: reverse transcriptase–PCR
